# Thermocatalytic Conversion of Plastics into Liquid Fuels over Clays

**DOI:** 10.3390/polym14102115

**Published:** 2022-05-23

**Authors:** Evgeniy S. Seliverstov, Lyubov V. Furda, Olga E. Lebedeva

**Affiliations:** 1 Department of Biology, Institute of Pharmacy, Chemistry and Biology, Belgorod State National Research University, 308015 Belgorod, Russia; seliverstov.evgeniy.s@gmail.com; 2 Department of General Chemistry, Institute of Pharmacy, Chemistry and Biology, Belgorod State National Research University, 308015 Belgorod, Russia; furda@bsu.edu.ru

**Keywords:** secondary raw materials, plastics, fuel, catalysts, clays, clay minerals, thermocatalytic conversion

## Abstract

Recycling polymer waste is a great challenge in the context of the growing use of plastics. Given the non-renewability of fossil fuels, the task of processing plastic waste into liquid fuels seems to be a promising one. Thermocatalytic conversion is one of the methods that allows obtaining liquid products of the required hydrocarbon range. Clays and clay minerals can be distinguished among possible environmentally friendly, cheap, and common catalysts. The moderate acidity and the presence of both Lewis and Brønsted acid sites on the surface of clays favor heavier hydrocarbons in liquid products of reactions occurring in their pores. Liquids produced with the use of clays are often reported as being in the gasoline and diesel range. In this review, the comprehensive information on the thermocatalytic conversion of plastics over clays obtained during the last two decades was summarized. The main experimental parameters for catalytic conversion of plastics according to the articles’ analysis, were the reaction temperature, the acidity of modified catalysts, and the catalyst-to-plastic ratio. The best clay catalysts observed were the following: bentonite/spent fluid cracking catalyst for high-density polyethylene (HDPE); acid-restructured montmorillonite for medium-density polyethylene (MDPE); neat kaolin powder for low-density polyethylene (LDPE); Ni/acid-washed bentonite clay for polypropylene (PP); neat kaolin for polystyrene (PS); Fe-restructured natural clay for a mixture of polyethylene, PP, PS, polyvinyl chloride (PVC), and polyethylene terephthalate (PET). The main problem in using natural clays and clay minerals as catalysts is their heterogeneous composition, which can vary even within the same deposit. The serpentine group is of interest in studying its catalytic properties as fairly common clay minerals.

## 1. Introduction

The last few centuries have been marked by the rapid development of mankind. The obvious benefits that it brought were accompanied by new, serious anthropogenic challenges. One of them was the emergence in the 1950s of new synthetic materials—plastics. The main ingredient of plastic are polymers, such as polyolefins (with commercially dominant polyethylene and polypropylene) possessing the general formula (CH_2_CHR)_n_ where R is an alkyl group, polystyrene ((C_6_H_5_CH = CH_2_)_n_), polyvinyl chloride ((C_2_H_3_Cl)_n_), etc. Disposable tableware, containers, packaging, and many other plastic products have firmly entered our everyday life, but their uncontrolled disposable use has created a huge threat to the environment. Nature was not ready for this amount of difficult-to-recycle material in a very short time, and despite recent reports of microorganisms across the globe adapting themselves to plastic degradation [1], it is still our urgent responsibility to resolve this problem.

One of the promising solutions is the conversion of plastic waste into liquid fuels. With a catalyst sufficiently selective to produce a mixture of hydrocarbons with an expected carbon number range, it would be possible to obtain liquid products with a composition similar to that of fuels such as gasoline and diesel. Since the production of various catalysts is often accompanied by environmental pollution, a complex preparation process, and, as a result, a high price of the final product, the catalysts must also comply with the principles of green chemistry and have a low cost.

Solid acid catalysts are among the most effective in the catalytic conversion of plastics. The process of the thermocatalytic transformation using these catalysts mostly depends on the presence of acid sites [2] and the number and size of catalyst pores [3]. Many works, including those of our group, are devoted to the use of synthetic aluminosilicates [4,5]. In particular, the synthesis and application of specific nanosponges of solid acids, named “acidic aluminosilicates”, should be mentioned as one of the latest achievements [6]. 

Microporous zeolite catalysts have high acidity active sites, which makes them able to split carbon-carbon bonds [2]. However, the small pore size of zeolites restricts the access of large molecules to acid sites located inside the channels. The presence of this steric factor leads to a higher yield of gases and a relatively high concentration of branched hydrocarbons among the degradation products since the contact of the polymer chain occurs mainly with the outer surface of the zeolite. In addition, a significant number of solid degradation products are formed on the surface and inside the pores of the catalyst. This leads to zeolite pore closure and catalytic deactivation. In the process of polymer cracking, these catalysts provide high selectivity for gaseous products.

Clays are moderately acidic, so reactions occurring within their pores favor the transformation of heavier hydrocarbons into liquid products than those of zeolites [7].

Liquids produced from plastic waste with the use of clay catalysts are often reported as being in the gasoline and diesel range [8]. Moreover, the layered structure of clays allows the formation of a porous network by alternating plates with so-called pillars (three-dimensional species as interlayer cations), thus creating interconnected micropores larger than those of zeolites [7]. Such materials demonstrate high stability and the possibility of reuse during heating to high temperatures [9].

Several reviews have been published on the topic of catalytic pyrolysis of plastics and the search for low-cost catalysts recently [10,11]. However, the works devoted to using clay-based catalysts were covered there briefly, and they did not include all available research on the activity of clay minerals of different groups. Peng et al. mentioned only montmorillonites and their analogs [10], while Fadillah et al. considered a few articles on kaolin and bentonite activity [11].

The task of collecting comprehensive information on the thermocatalytic conversion of plastics over clays obtained by different authors during the last two decades was set during the literature analysis. Works published in the last five years (2017–2021, including 2022) have been highlighted in bold in the tables to focus the attention on the latest results.

Natural materials containing clay minerals (hydrous aluminum silicates with variable amounts of cations) originated from natural sites or synthesized are designated as «clays» in the context of the present review. The application of pristine clays is rather rare. Usually, the clays are modified by different treatments. Modified clays are also the subjects of this review.

The following types of plastic materials are designated merely by the abbreviations to simplify the perception in the following text below: high-density polyethylene (HDPE), medium-density polyethylene (MDPE), low-density polyethylene (LDPE), polypropylene (PP), polystyrene (PS), polyethylene terephthalate (PET), polyvinyl chloride (PVC), ethylene-vinyl acetate (EVA).

## 2. Nature of Catalytic Activity of Clays

**Figure 1:**
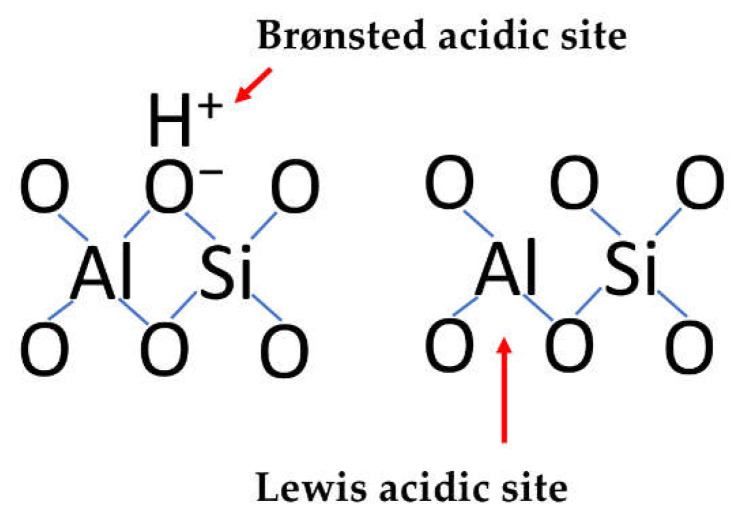
Acidic sites of clays.

Clays belong to solid acids. They have both Lewis and Brønsted acid sites (Figure 1) [7].

The acidic sites are comparatively strong (H_0_ typically quoted in the range from −5.6 to −8.2), though not as strong as the zeolite ones [7]. All the clays being aluminosilicates, the nature of the active sites is essentially the same for all types of clays. It is porosity that defines the specific features of different clays. Microporosity depends on the crystallographic structure of the material. There is an additional factor influencing the porosity of the clays. Their part-amorphous nature provides mesoporosity over a wide range of pore sizes.

Original clays in cationic forms usually contain an insufficient number of acidic sites since the sites involve protons (Figure 1). Only cationic deficient samples of clays demonstrate catalytic activity in the reactions of the acid-base type. Generally, acidic activation is necessary for obtaining catalytically active clays. The conditions of acidic treatment are often crucial for the efficiency of the clay catalysts.

## 3. Kaolin Group Catalytic Activity

The kaolin group is represented by layered phyllosilicate minerals with the chemical composition Al_2_Si_2_O_5_(OH)_4_. The layers of these clay minerals consist of corner-sharing tetrahedra and edge-sharing octahedra. Tetrahedra are formed by silicon atoms, and octahedrons are constructed from aluminum atoms. The way the layers are stacked and the nature of the material between the layers distinguishes the individual minerals (kaolinite, dickite, halloysite, and nacrite, sometimes also serpentine subgroup) in the group [12]. Rocks rich in kaolinite are thus called kaolin.

Kaolin-based catalysts are the most commonly mentioned among the articles on clay catalysts for the conversion of plastics into liquid fuels due to the abundant availability of natural kaolin. All results from work on kaolin clay catalysts are presented in Table 1. The symbol + is used when a mixture of polymers is described in the publication.

In the work of Liu et al., natural kaolinite-containing clay had no acidic sites and did not show any effect on the degradation temperature of HDPE [13]. However, it produced liquid oil with a yield of 16 wt%, a number of gaseous products much smaller than that of thermal degradation with a yield of 3.4 wt%, and a number of alkanes larger than that of olefins. The authors concluded that a clay catalyst was favorable for the enhancement of the intermolecular hydrogen transfer reaction and inhibition of the β-scission reaction of radicals compared to thermal degradation, which was related to the hydrogen bonds from the layer structure and large mesopores.

It appears that the best result for the high-density polyethylene degradation was obtained by Kumar and Singh using the response surface methodology (RSM) [14]. RSM allowed a reduction in the number of costly experiments by selecting the right experimental conditions. It can be considered a promising method for the evaluation of selected experimental variables in the planning step of such experiments. The optimized values of experimental variables were 450 °C, 0.341, and 1:4 for reaction temperature, the acidity of the catalyst, and the catalyst-to-waste HDPE ratio, respectively, to produce a maximum liquid fuel yield of 78.7%.

Luo et al. studied the possibility of reusing the kaolin catalyst. They found that the yield of aromatic hydrocarbons increases with the reuse of the catalyst, which is associated with an increase in particle size [16]. The optimal size of kaolin particles was investigated in the work of Erawati et al., where it was established as 7.5 × 6.5 cm [19].

The influence of non-inert reaction conditions on products of thermocatalytic conversion was shown by Uzair et al. [23]. Alcohols and ketones were formed due to oxidative cracking of PP.

The work of Auxilio et al. proved that catalyst surface Lewis acidity was critical for hydrocarbon fraction selectivity, and higher acidity favors gasoline formation, while low to mild acidity favors diesel formation [31]. They found that mesopore volume was a crucial factor in avoiding catalyst coking because the small mesopore volume favored high overall coke formation. In addition, the authors stated that using a pellet form catalyst was advantageous over powder form to avoid large pressure drops in the reactive distillation column.

In summarizing, the highest liquid yields described in the articles concerned with kaolin catalysts for different plastics are 78.7 wt% for HDPE over nitric acid-treated kaolin [14], 99.82 wt% for LDPE on neat kaolin powder [19], 92 wt% for PP on sulfuric acid-treated kaolin [25], and 96.37 wt% for PS over pristine kaolin [29]. Quite expectably, the most efficient catalysts were obtained using acidic treatment, which led to the generation of a sufficient number of acidic sites.

## 4. Smectite Group Catalytic Activity

Members of the smectite group include the dioctahedral minerals (montmorillonite, beidellite, and nontronite) and the trioctahedral minerals (hectorite, saponite, and sauconite). The basic structural unit of these clay minerals is a layer consisting of two inward-pointing tetrahedral sheets with a central alumina octahedral sheet [33]. The clay consisting mostly of montmorillonite is called bentonite, but in commerce, this term can be used in a more general way to refer to any swelling clay composed mostly of minerals from the smectite group.

The bentonite- and pure montmorillonite-based catalysts are the most commonly occurred besides smectite catalysts for plastic transformation. There are a few articles devoted to the use of saponite and beidellite. All results from the work on smectite clay catalysts are presented in Table 2.

Pillared bentonite clays were selective to cracking HGO/HDPE in light hydrocarbons (C_10_–C_23_) and produced a light linear hydrocarbon content 63% higher than that produced with zeolite [35].

The work of Elordi et al. draws attention to the result obtained by the authors that pristine bentonite does not demonstrate catalytic activity at 500 °C in a conical spouted bed reactor in the continuous regime (1 g min^–1^ of HDPE is fed) [34]. However, agglomeration of 50 wt% bentonites with spent fluid catalytic cracking catalyst (FCC) allows the thermal cracking of the initial macromolecules in the mesopores of the clay until they reach the spent FCC particles.

Gobin and Manos noted that even if clays were less active than zeolites, they could fully degrade the polymer [47]. In this work, the authors used montmorillonite (Zenith-N), saponite (with a small number of impurities, mainly sepiolite), and their pillared derivatives. They showed enhanced liquid formation and lower coke formation. Regenerated pillared clays offered practically the same performance as fresh samples, but their original performance deteriorated after the removal of the formed coke.

Summing up, the highest liquid yields described in the articles for smectite catalysts for different plastics are 100 wt% for HDPE over bentonite (50 wt%)/spent fluid catalytic cracking catalyst (FCC) [34], about 70 wt% for MDPE over acid-restructured montmorillonite catalyst [45], 87.6 wt% for LDPE over pelletized bentonite [8], 92.76 wt% for PP over Ni/acid-washed bentonite clay [40], and 88.78 wt% for PS on acid-treated bentonite-based catalyst [39]. The full conversion of the HDPE in the case of using the FCC was achieved according to step-by-step reactions where on the first step, the thermal cracking of the initial macromolecules occurred in the mesopores of the bentonite until they reached the spent FCC catalyst particles.

## 5. Other Clay Minerals’ Catalytic Activity

The variety of clay minerals is not limited to the above-mentioned two groups. Only a few examples of studying the catalytic activity of other clay minerals (sepiolite, vermiculite, talc, and pyrophyllite) in relation to plastics were found (Table 3).

Interestingly, in the case of talc, its catalytic activity was revealed by chance [57]. Talc is often a filler in polypropylene that increases its stiffness. The product yields and compositions from pure PP and PP with fillers showed a significant difference, indicating a higher degree of degradation for PP with fillers, most likely resulting from the fillers acting as a catalyst. It produced a much higher gas yield (76.3%) and a negligible wax yield.

Khan and Hussain also reported the catalytic activity of talk (French chalk, as mentioned in the work) [18]. They indicated that the products of the pyrolysis of the French chalk catalyzed reactions contain no wax and give a greater proportion of the oil as well as gaseous products.

The results obtained for sepiolite show that, despite the low “nominal” catalytic activity of this clay, it has enough catalytic properties to decrease the temperature of decomposition of PE and PP [55]. However, the steric effects related to the substituents of PS and EVA cancel this catalytic behavior. Experiments performed in an oxidizing atmosphere showed that there was no noticeable decrease in the temperature that may be related to the presence of the clay.

The Co/Verm and Ni/Verm catalysts in the work of Chen et al. had higher selectivity for fractions with a carbon number greater than C_13_ [56]. Organic Verm and CoNi/Verm catalysts had higher selectivity for fractions with a carbon number less than C_13_. Due to the interaction between acidity and texture properties, the modified catalyst could produce a large amount of diesel oil, a distillate from petroleum products, and H_2_ in natural gas products.

The acid-treated pyrophyllite catalyst also showed good catalytic performance for the degradation of PS [32]. Compared to thermal degradation, catalysts showed much higher selectivity for ethylbenzene and much lower production of C_16_–C_21_ (8.45%).

To sum it up, the most promising liquid yields were obtained by degradation of LDPE on talc (91 wt%) [18] and PS on pyrophyllite (88.3 wt%) [32]. Similar to the cases for the above-described kaolin acidic treatment allows reaching the highest efficiency in plastic conversion.

## 6. Catalytic Activity of Mixed Natural Clays

Some works were focused on uncharacterized mixed clays from different fields (Table 4).

For example, Filip et al. investigated the thermal degradation processes at 420 °C of a plastic waste mixture (PS + PET + PVC) in the absence and presence of two types of natural Romanian clay catalysts [65]. The GC-MS results showed that the liquid fractions contained mainly monoaromatic compounds. The highest amounts of styrene come from thermal degradation of PS, which was the major component in the plastic mixture. The Vadu Crişului clay catalyst has been found as the most efficient catalyst for the thermal degradation of a plastic mixture.

In summarizing, the highest liquid yields were obtained for LDPE on Fuller’s earth catalyst (91 wt%) [18]; PP on natural clay mineral from Indonesia impregnated with LaFeO_3_ nanoparticles (88.8 wt% on the 5th cycle) [60]. It should be stressed that in the latter case, the yield growth was provided by the specific efficient promoter—lanthanum ferrite. This significant distinction of the catalyst attracts attention to the perspectives of non-acidic modification of clays.

## 7. Conclusions and Perspectives

The results of numerous researches give evidence that the main experimental parameters for thermocatalytic conversion of plastics were the reaction temperature, the acidity of modified catalysts, and the catalyst-to-plastic ratio. By varying the parameters, one can achieve an essential increase in the yield of the liquid hydrocarbons in the process of plastic conversion.

The best clay and clay-based catalysts with the highest liquid yields among works described in this review for each of the plastic were the following: bentonite/spent FCC for HDPE; acid-restructured montmorillonite for MDPE; neat kaolin powder for LDPE; Ni/acid-washed bentonite clay for PP; neat kaolin for PS; Fe-restructured natural clay for a mixture of PE, PP, PS, PVC, and PET. It can be seen that the modification of clay catalysts (acid-washing or pillaring) in some cases helps to achieve a higher yield of the liquid fraction. However, some pure clays and clay minerals are also showing excellent catalytic activity.

The principal problem in using natural clays and clay minerals as catalysts is their heterogeneous composition, which can vary even within the same deposit. Therefore, studies on their use should begin with a thorough characterization of the samples used—their elemental composition, particle size and porosity, acidity, etc. Otherwise, the main rule of reproducibility of scientific results is violated, and works using the same clays and clay minerals can obtain drastically different results, leading to confusion.

Despite the availability of well-studied catalysts based on kaolin, bentonite, and montmorillonite, many other clay minerals remain poorly studied as prospective catalysts. For instance, the serpentine group (often combined with kaolin in the kaolin-serpentine group) is a set of common rock-forming hydrous magnesium iron phyllosilicate ((Mg,Fe)_3_Si_2_O_5_(OH)_4_) minerals commonly found in serpentinite rocks. Serpentinite has not been used directly as a catalyst but has shown very interesting results as a precursor to producing active catalysts (i.e., the intercalation of serpentine with the alkaline metals gave rise to the basic catalysts for the production of biodiesel). Thus, the serpentine group is of interest in studying its catalytic properties as fairly common but not well-studied clay minerals. Minerals of the chlorite, illite, and halloysite groups also deserve a separate investigation.

Another promising direction of future studies is clay activation and modification. Various examples of modifications thus far applied by different authors cannot be considered a comprehensive list of possible treatments. Some well-known methods of clay activation, such as UV-irradiation, mechanical treatment, and especially chemical promotion, are still of interest.

## Figures and Tables

**Table 1:** Publications on the conversion of different plastics over clay minerals from kaolin group catalysts.

Catalyst	Plastic	Temperature, °C	Highest Liquid Yield, wt%	Specific Results	Reference
Kaolinite-containing natural clay	HDPE	478	16	Catalyst produced more alkanes than olefins in both gaseous and liquid oil products.	[13]
Kaolin and its modifications With CH_3_COOH, HCl, H_3_PO_4_, HNO_3_, and NaOH	HDPE	450	78.7	The liquid fuel consisted of petroleum products range hydrocarbons (C_10_–C_25_).	[14]
Kaolin	LDPE	450	79.5	The oil consists of paraffins and olefins with a predominance of C_10_–C_16_ components.	[15]
**Kaolin**	**LDPE**	**600**	**about 75**	**The first addition of kaolin gives aliphatic compounds and C_6_–C_20_ aromatics (90–95%).**	[16]
**75% kaolinite with 25% bentonite**	**LDPE**	**580**	**74.45**	**High yield of paraffins (70.62%). The percentage of aromatics was 5.27%.**	[17]
China clay (kaolinite)	LDPE	300	84	Components with a boiling point of 125–180°C were identified as alkanes, alkenes, and aromatics.	[18]
**Kaolin**	**LDPE**	**450**	**99.82**	**The highest percentage component is heptane.**	[19]
**Al-substituted Keggin tungstoborate/kaolin composite**	**LDPE**	**295**	**84**	**During the catalytic cracking 70 mol.% of gasoline range hydrocarbons were produced.**	[20]
**tungstophosphoric acid/kaolin composite**	**LDPE**	**335**	**81**	**A high content of benzene-like hydrocarbons (C_11_–C_14_).**	[21]
**Ahoko kaolin**	**PP**	**450**	**79.85**	**Liquid products with properties comparable to conventional fuels (gasoline and diesel).**	[22]
Hydrochloric acid/kaolin composite	PP	470	71.9	The condensable hydrocarbons contain dominantly alkanes and alkenes in the range C_6_–C_12_.	[23]
Commercial-grade kaolin clay	PP	450	89.5	Contains olefins, aliphatic, and aromatic hydrocarbons in the oil comparable with liquid fossil fuels.	[24]
Commercial-grade kaolin clay and kaolin treated with sulfuric acid	PP	500	92 (acid-treated), 87.5 (neat kaolin)	The oil from the neat kaolin—C_10_–C_18_ products, from the acid-treated kaolin—mainly C_9_–C_13_.	[25]
Kaolin	PP	500	87.5	Fuel properties are identical to the different petroleum fuels.	[26]
Neat kaolin and kaolin treated with hydrochloric acid	PP	400–500	71.9	The highest yield of liquid hydrocarbons was achieved with kaolin clay treated with 3M HCl.	[27]
Kaolin	PP/vaseline (4.0 wt%)	520	52.5	The gasoline—32.77%, diesel—13.59%, residue—6.14%	[28]
**CuO/kaolin and neat kaolin**	**PS**	**450**	**96.37 (neat kaolin), 92.48 (CuO/kaolin)**	**The oil contained aromatic hydrocarbons, but from CuO/kaolin—85% C_10_H_8_ and ~13% C_8_H_8_.**	[29]
**Zeolite-Y + metakaolin + aluminum hydroxide + sodium silicate all synthesized from kaolin**	**HDPE + LDPE + PP + PS + PET**	**350**	**46.7**	**Catalyzed fuel samples consist of 93% gasoline and 7% diesel fraction.**	[30]
**Kaolin**	**Virgin HDPE, HDPE waste and mixed plastic waste**	**425**	**79**	**The catalyst was the most selective in producing diesel, which yielded 63%.**	[31]
Halloysite treated with hydrochloric acid	PS	450	90.2	Aromatic compounds of more than 99%. The main product is styrene (58.82%).	[32]

**Table 2:** Publications on the conversion of different plastics over clay minerals from smectite group catalysts.

Catalyst	Plastic	Temperature, °C	Highest Liquid Yield, wt%	Specific Results	Reference
Bentonite (50 wt%)/spent fluid catalytic cracking catalyst (FCC)	HDPE	500	100	High yields of gasoline C_5_–C_11_ (50 wt%) The yield of C_12_–C_20_ hydrocarbons—8–10 wt%.	[34]
**Pillared bentonite (PILC) intercalated with Fe or Al**	**HDPE and heavy gas oil (HGO)**	**500**	**>80**	**The oil from the Fe-PILC-Fe-300 catalyst was more similar to the standard diesel.**	[35]
Bentonite (Gachi clay)	LDPE	300	77	Olefin and paraffin hydrocarbons.	[36]
**South Asian clay classified as bentonite and** **montmorillonite impregnated with nickel NPs**	**LDPE and post-consumer polybags**	**350**	**79.23 (LDPE), 76.01 (poly-bags)**	**The final products are in the range of gasoline, kerosene, and diesel.**	[37]
**Bentonite thin layer loaded with MnO_2_ nanoparticles (NPs)**	**PP**	**750**	**Parameters were designed to get off the liquid**	**The complete decomposition of plastics with the formation of gases (methane and hydrogen) and coke.**	[38]
**Bentonite treated with 0.5M hydrochloric acid**	**PS**	**400**	**88.78**	**The obtained liquid contains styrene. Toluene and benzene were the major components.**	[39]
Acid-washed bentonite clay (AWBC), Zn/AWBC, Ni/AWBC, Co/AWBC, Fe/AWBC, Mn/AWBC	PP, HDPE	300 for PP and 350 for HDPE	AWBC (PP 68.77, HDPE 70.19), Ni/AWBC (PP 92.76, HDPE 62.07), Co/AWBC (PP 82.8, HDPE 69.31), Fe/AWBC (PP 82.78, HDPE 71.34), Mn/AWBC (PP 80.4, HDPE 81.07), Zn/AWBC (PP 82.50, HDPE 91)	Co/AWBC/PP (mainly olefins and naphthenes) and Zn/AWBC/HDPE (mainly paraffins and olefins) were the most effective.	[40]
H_2_SO_4_-activated bentonite (synthesized)	PP + HDPE	328	79	The hydrocarbon oil.	[41]
**A mixture of nature bentonite and zeolite (70:30)**	**PP, PET**	**400**	**78.42 (PP), 72.38 (PP + PET)**	**The number of C_3_–C_10_ compounds increased.**	[42]
**Pelletized bentonite**	**PS, PP, LDPE, HDPE**	**500**	**88.5 (PS), 90.5 (PP), 87.6 (LDPE), 88.9 (HDPE)**	**PS—95% aromatic hydrocarbons; PP, LDPE, and HDPE—aliphatic hydrocarbons; LDPE, and HDPE—diesel fuel (96% similarity); PS—gasohol 91.**	[8]
**Calcium bentonite**	**PP, LDPE, HDPE, PP + LDPE + HDPE**	**500**	**88.5 (PP), 82 (LDPE), 82.5 (HDPE) 81 (PP + LDPE + HDPE)**	**The oil contained only a mixture of hydrocarbons and has matching fuel properties as that of fossil fuel. Mixed plastics—C_10_-C_28_.**	[43]
**Pillared bentonite (Al-PILC, Fe-PILC, Ti-PILC, Zr-PILC)**	**HDPE + PS + PP + PET**	**300–500**	**68.2 (Al-PILC), 79.3 (Fe-PILC), 62.8 (Ti-PILC), 62.1 (Zr-PILC)**	**80.5% diesel fraction was observed in presence of Fe-PILC.**	[7]
**Fe/Al pillared montmorillonite mixed with an acid Commercial bentonite as a binder**	**HDPE**	**600**	**About 40**	**The catalyst gave high yields of waxes, particularly rich in diesel hydrocarbon range (C_11_–C_21_).**	[44]
commercial acid-restructured montmorillonite and Al- and Fe/Al-pillared derivative	MDPE	300	About 70	The clay-based catalysts gave higher yields of liquid products in the C_15_–C_20_ range. Clay catalysts produce liquid hydrocarbons in the gasoline and diesel range.	[45]
**Al_2_O_3_-pillared montmorillonite (calcium rich)**	**LDPE**	**430**	**70.2**	**Hydrocarbons from C_5_ to C_13_.**	[46]
Montmorillonite (Zenith-N) and a pillared derivative	LDPE	427	68 (montmorillonite), 75 (pillared derivative)	Clays showed enhanced liquid formation due to their mild acidity.	[47]
Al-pillared montmorillonite (Al-PILC), and regenerated samples	LDPE	360	72 (Al-PILC), 68 (regenerated sample)	These products were in the boiling point range of motor engine fuels.	[48]
Montmorillonite (Zenith-N) and a pillared derivative	LDPE	360	75 (montmorillonite), 76 (pillared derivative)	These products were in the boiling point range of gasoline.	[49]
Ionically bonding macrocyclic Zr-Zr complex to montmorillonite	PP	300–400	-	A low molecular weight waxy product with paraffin wax characteristics was obtained.	[50]
Untreated and Al-pillared montmorillonite clay	PS	400	83.2 (untreated clay), 81.6 (Al-pillared clay)	Styrene was the major product, and ethylbenzene was the second most abundant one in the liquid product.	[51]
Four different types of montmorillonites: K5, K10, K20, K30	LDPE, PP, and the municipal waste plastics	begins at 250 for mK5 (LDPE), 210–435 for mK20 (PP)	Data not presented	The catalytic degradation products contain a relatively narrow distribution of light hydrocarbons.	[52]
Organically modified montmorillonite/Co_3_O_4_	PP + HDPE + PS	700	59.6	The catalyst promoted the degradation of mixed plastics into light hydrocarbons and aromatics.	[53]
**cloisite 15 A as a natural montmorillonite modified with a quaternary ammonium salt**	**Industrial grade of HDPE, which was a copolymer with 1-hexene (1.5 wt%) as comonomer**	**473.7**	**Data not presented**	**It was found that the nano clay reduces the temperature at a maximum degradation rate.**	[54]
Commercial acid-restructured saponite and Al- and Fe/Al-pillared derivatives	MDPE	300	About 70	The clay-based catalysts gave higher yields of liquid products in the C_15_–C_20_ range. Clay catalysts produce liquid hydrocarbons in the gasoline and diesel range.	[45]
Saponite, with a small number of impurities, mainly sepiolite and a pillared derivative	LDPE	427	83 (saponite), 82 (coked pillared derivative)	Clays showed enhanced liquid formation due to their mild acidity.	[47]
Al-pillared saponite and regenerated samples	LDPE	360	72 (pillared saponite), 67 (regenerated sample)	These products were in the boiling point range of motor engine fuels.	[48]
Saponite and a pillared derivative	LDPE	360	68 (saponite), 72 (pillared derivative)	These products were in the boiling point range of gasoline.	[49]
Commercial acid-restructured beidellite and Al- and Fe/Al-pillared derivatives	MDPE	300	About 70	The clay-based catalysts gave higher yields of liquid products in the C_15_–C_20_ range. The catalysts produce liquid hydrocarbons in the gasoline and diesel range.	[45]

**Table 3:** Publications on the conversion of different plastics over sepiolite, talc, pyrophyllite, and vermiculite catalysts.

Catalyst	Plastic	Temperature, °C	Highest Liquid Yield, wt%	Specific Results	Reference
Commercial sepiolite	PE, PP, PS, EVA	432.65 (PE), 401.65 (PP), 449.75 (PS), 459.85 (EVA)	Data not presented	Clay reduces the decomposition temperatures of PE and PP. However, steric effects associated with the PS and EVA substituents nullify this catalytic behavior.	[55]
**Tetraethyl silicate modified vermiculite, Co, and Ni intercalated vermiculite**	**PP + PE**	**300-480**	**80.6 (organic vermiculite), 73.2 (Co/verm), 70.7 (Ni/verm), 73.9 (Co/Ni/verm)**	**The obtained liquid is mainly composed of C_9_–C_12_ and C_13_–C_20_.**	[56]
Talc (French chalk)	LDPE	300	91	Components with a boiling point of 125–180°C were identified as alkanes, alkenes, and aromatics.	[18]
**Talc (plastic filler)**	**PP**	**620**	**About 23**	**The liquid product contained a higher aromatic content (57.9%) and a lower n-alkene content (5.8%).**	[57]
Pyrophyllite treated with hydrochloric acid	PS	450	88.3	The catalysts showed selectivity to aromatics over 99%. Styrene (63.40%) is the major product, and ethylbenzene is the second-most abundant one (6.93%).	[32]

**Table 4:** Publications on the conversion of different plastics over clays from different fields.

Catalyst	Plastic	Temperature, °C	Highest Liquid Yield, wt%	Specific Results	Reference
**Acid-activated fire clay (Pradeep Enterprises, Ajmeri Gate, Delhi)**	**HDPE**	**450**	**41.4**	**The identified compounds were mainly paraffins and olefins with a carbon number range of C_6_–C_18_.**	[58]
Indian Fuller’s earth (Multan clay)	LDPE	300	58.33	The obtained liquid contained olefin, paraffin, and aromatic hydrocarbons. Light naphtha—15%, heavy naphtha—35%, middle distillate—60%.	[59]
Fuller’s earth	LDPE	300	91	Components with a boiling point of 125-180°C were identified as alkanes, alkenes, and aromatics.	[18]
**Natural clay mineral (Indonesia) with LaFeO_3_ NPs**	**PP**	**460–480**	**88.8 (5th cycle)**	**The liquid fraction: alkanes (44.70%), alkenes (34.84%), cyclo-alkanes (9.87%), cyclo-alkenes (3.07), branched-chain alkanes (2.42%), branched-chain alkenes (0.88%).**	[60]
**natural clay with kaolinite, hematite, smectite, quartz**	**PS**	**410**	**86.68**	**Fuel properties of the liquid fraction obtained showed a good resemblance with gasoline and diesel oil.**	[61]
**Red clay (Auburn, Alabama, USA)**	**PS and LDPE (co-pyrolysis with a lignin)**	**500, 600, 700, 800**	**data not presented**	**The carbon yield of a lignin-derived compound, guaiacol, increased during co-pyrolysis of lignin with LDPE, and PS with red clay as a catalyst.**	[62]
Shwedaung clay, Mabisan clay	HDPE + LDPE + PS + PP + PET	210-380	65.81 (Shwedaung clay), 67.06 (Mabisan clay)	Fuel can be used internal combustion engine after distillation. Char can be used as solid fuel.	[63]
**Fe-restructured clay (Fe-RC)**	**PE + PP + PS + PVC + PET**	**450**	**83.73**	**High selectivity for the C_9_–C_12_ and C_13_–C_19_ oil fractions, which are the major constituents of kerosene and diesel fuel.**	[64]
Romanian natural clays: Vadu Crişului clay and Lugoj clay	PS + PET + PVC	420	62.18 (Vadu Crişului clay), 54.98 (Lugoj clay)	The liquid products contained monoaromatic compounds such as styrene, toluene, ethylbenzene, or alpha-methylstyrene.	[65]
